# Silicon-Induced Mitigation of NaCl Stress in Barley (*Hordeum vulgare* L.), Associated with Enhanced Enzymatic and Non-Enzymatic Antioxidant Activities

**DOI:** 10.3390/plants11182379

**Published:** 2022-09-12

**Authors:** Muhammad Salim Akhter, Sibgha Noreen, Ume Ummara, Muhammad Aqeel, Nawishta Saleem, Muhammad Mahboob Ahmed, Seema Mahmood, Habib-ur-Rehman Athar, Mohammed Nasser Alyemeni, Prashant Kaushik, Parvaiz Ahmad

**Affiliations:** 1 Institute of Botany, Bahauddin Zakariya University, Multan 60800, Pakistan; 2 Department of Botany, The Islamia University of Bahawalpur, Rahim Yar Khan Campus, Rahim Yar Khan 64200, Pakistan; 3 State Key Laboratory of Grassland Agro-Ecosystems, School of Life Science, Lanzhou University, Lanzhou 730000, China; 4 Institute of Chemical Science, Bahauddin Zakariya University, Multan 60800, Pakistan; 5 Botany and Microbiology Department, King Saud University, Riyadh 11451, Saudi Arabia; 6 Independent Researcher, 46022 Valencia, Spain; 7 Department of Botany, GDC, Pulwama 192301, Jammu and Kashmir, India

**Keywords:** abiotic stress tolerance, antioxidant defense, *Hordeum vulgare*, lipid peroxidation, proline, PCA-biplot

## Abstract

Salt stress obstructs plant’s growth by affecting metabolic processes, ion homeostasis and over-production of reactive oxygen species. In this regard silicon (Si) has been known to augment a plant’s antioxidant defense system to combat adverse effects of salinity stress. In order to quantify the Si-mediated salinity tolerance, we studied the role of Si (200 ppm) applied through rooting media on antioxidant battery system of barley genotypes; B-10008 (salt-tolerant) and B-14011 (salt-sensitive) subjected to salt stress (200 mM NaCl). A significant decline in the accumulation of shoot (35–74%) and root (30–85%) biomass was observed under salinity stress, while Si application through rooting media enhancing biomass accumulation of shoots (33–49%) and root (32–37%) under salinity stress. The over-accumulation reactive oxygen species i.e., hydrogen peroxide (H_2_O_2_) is an inevitable process resulting into lipid peroxidation, which was evident by enhanced malondialdehyde levels (13–67%) under salinity stress. These events activated a defense system, which was marked by higher levels of total soluble proteins and uplifted activities of antioxidants enzymatic (SOD, POD, CAT, GR and APX) and non-enzymatic (α-tocopherol, total phenolics, AsA, total glutathione, GSH, GSSG and proline) in roots and leaves under salinity stress. The Si application through rooting media further strengthened the salt stressed barley plant’s defense system by up-regulating the activities of enzymatic and non-enzymatic antioxidant in order to mitigate excessive H_2_O_2_ efficiently. The results revealed that although salt-tolerant genotype (B-10008) was best adopted to tolerate salt stress, comparably the response of salt-sensitive genotype (B-14011) was more prominent (accumulation of antioxidant) after application of Si through rooting media under salinity stress.

## 1. Introduction

Soil salinity is a serious global issue confining agricultural production and creating great economic loss. It has been estimated that globally more than 33% agricultural and 20% of total irrigated land is affected by high salinity, which is escalating at the rate of 10% per annum [1]. Meanwhile, in Pakistan 4.5 million hectares of arable land is being affected only by salinity and causing severe damage to crop as well as soil health [2]. Saline soils pose detrimental effects on global agricultural production as a result of its direct impact on the plant’s biochemical and molecular processes [3,4] primarily by inducing water deficit conditions; and secondarily through ionic toxicity and nutrient disequilibrium in cellular compartments [5,6].

It has been estimated that 20% of crop yield got reduced by nutrient imbalanced and soil contamination caused by salinity stress [7]. Moreover, an excessive accumulation of salts promotes leakage of reactive oxygen species (ROS) within the cell, and damages lipids, proteins and DNA structures [8,9]. Peroxisomes are main organelles for ROS (H_2_O_2_, O^2−^, ^1^O_2_, OH·) production during β-oxidation, fatty acid metabolism, photorespiration and glycolic acid oxidation reactions [10,11]. The immediate feedback of plants to scavenge excessive ROS is the activation of a defense system by producing soluble proteins and antioxidant (SOD, CAT, POD, AsA, α-tocopherol, phenolics, proline) [12,13]. These endogenous antioxidants efficiently detoxify ROS without damaging the cellular compartments.

Although plants have the ability to scavenge excessive ROS, this capacity is still limited but can be enhanced by the application of different chemicals, nutrient supplements and growth regulators [14], i.e., growth regulators [14,15], nutrients [16], amino acids [17] and silicon [18,19] are used as a shotgun approach to augment resistance in plants against stressful conditions. Several studies have drawn special attention to the role of silicon as a shotgun approach in improving plant resistance against stressful conditions. It is ranked as the second abundant element on earth’s crust [20,21]. Its presence in plants tissues and soil varies depending on the type of plant species and ability to uptake from soil. Si absorption and transportation is a complex process that involves the influx and efflux of Si through transporters of the aquaporin family with specific selectivity properties. It is absorbed by plant roots only in the form of Si(OH)_4_ through Si (*LSi1* and *LSi2*) transporters via apoplastic pathway [22,23].

The exogenous application of Si helps plants to mitigate the toxic effects of salinity by maintaining plant’s water relation [24], balanced Na^+^ and K^+^ levels [25] and boosted antioxidant response in different plant species as barley [26], rice [27], tomato [28], wheat [29] and maize [30]. Rooting the media application of Si helped to improve the plant’s growth and yield by effective detoxification of ROS. The supplement of Si has a slight edge over other exogenous application due to its high abundance and only a small amount of soluble silicon can alleviate salt tolerance and improve growth by modulating chlorophyll content and photosynthetic characteristics Therefore, it is need of time to understand the role of Si to understand the biochemical and antioxidant response of barley crop under salinity stress, to be used for better growth and productivity.

## 2. Results

### 2.1. Plants Vegetative Growth

Vegetative growth of shoot and root (biomass and lengths) of barley genotypes showed significant effect of 200 mM NaCl and 200 ppm Si. The data showed that Si application increased shoot length (22% and 18%) and root length (20% and 17%), shoot fresh weight (18% and 11%), shoot dry weight (23% and 19%), root fresh weight (10% and 17%) and root dry weight (11% and 10%) in B-10008 and B-14011, respectively, as compared to control plants. It has been observed that 200 mM NaCl caused a reduction in: shoot length (27% and 60%); root length (19% and 57%); shoot fresh weights (30% and 78%); shoot dry weights (38% and 73%); root fresh weight (30 and 85%); and root dry weight (32% and 87%) in B-10008 and B-14011, respectively, However, the application of 200 ppm Si reduced the effect of salt (200 mM NaCl) stress by enhancing: shoot length (17% and 15%); root length (22% and 11%); shoot fresh weights (15% and 16%); shoot dry weights (21% and 14%); root fresh weight (36% and 33%); and root dry weight (37% and 31%) in B-10008 and B-14011, respectively, as compared to respective saline treatments (Table 1).

### 2.2. Total Soluble Proteins (TSP)

It has been observed that the presence of NaCl in rhizosphere considerably (*p* < 0.001) boosted the synthesis of TSP in leaves (22%) and roots (up to 30%) compared to control plants (Table 2). The exogenously applied to Si through rooting media to barley plants increases TSP contents (6–11%) when compared to control plants, while this increase was 7–47% under salinity stress. However, B-10008 accumulated higher leaf (17%), while B-14011 accumulated higher root (44%) TSP contents after rooting application of Si (200 ppm) under salinity stress (Table 2).

### 2.3. Total Free Amino Acids (TFAA)

The results revealed that salinity stress significantly enhanced TFAA B-14011 (73% and 103%) as compared to B-10008 (18% and 24%) in leaves and roots, respectively, as compared to control treatment (Table 2). However, TFAA contents in leaves and roots were reduced from 6% (B-10008) to 13% (B-14011) and 6%, (B-10008) to 15% (B-14011), respectively, after Si application when compared to saline treatment alone (Table 2).

### 2.4. H_2_O_2_ and MDA Content

The data (Table 2) exhibited that when compared to control conditions the leaf and root’s H_2_O_2_ contents were increased under a salty environment (200 mM NaCl). Under salt stress, the maximum increase in leaf (372%) and roots (152%) H_2_O_2_ contents were observed in B-14011 and B-10008, respectively. While the application of 200 ppm Si detoxified the harmful effect of H_2_O_2_ in leaves (36%) and roots (42%) under salinity stress as compared to control. The MDA level is the indicator of lipid peroxidation and was increased with the increase in ROS (H_2_O_2_). The MDA was more significantly enhanced in roots (67%) and leaves (29%) of sensitive genotype (B-14011) while Si application tends to lower MDA contents in leaves and roots to 40% and 45%, respectively, under salinity stress when compared to their respective control plants (Table 2). The degree of H_2_O_2_ detoxification and MDA contents was more pronounced in leaves of B-10008, while the roots of B-14011 showed maximum decline with Si application under salinity stress when compared to the counterpart barely genotype.

### 2.5. Activity of Enzymatic Antioxidants

Elimination of ROS produced during oxidative stress is a natural phenomenon in plants. As the plants counter with excessive ROS (O_2_^−^, OH·, H_2_O_2_) they start synthesizing antioxidants to minimize its harmful effect. Under salinity stress (200 mM NaCl) the activity of enzymatic antioxidant was augmented in the leaves and root of barley genotypes. Comparatively the activities antioxidants, i.e., SOD (27%), POD (32%), APX (22%) and GR (45%) in leaves and the activities of SOD (38%), APX (46%), CAT (33%) and GR (35%) in roots of tolerant genotype (B-10008) was higher under salt stress as compared to control counterparts. Whereas in salt sensitive genotype, CAT (88%) and POD (75%) in leaves and POD (68%) activities in roots were higher under salinity stress when compared to control plants.

**Figure 1:**
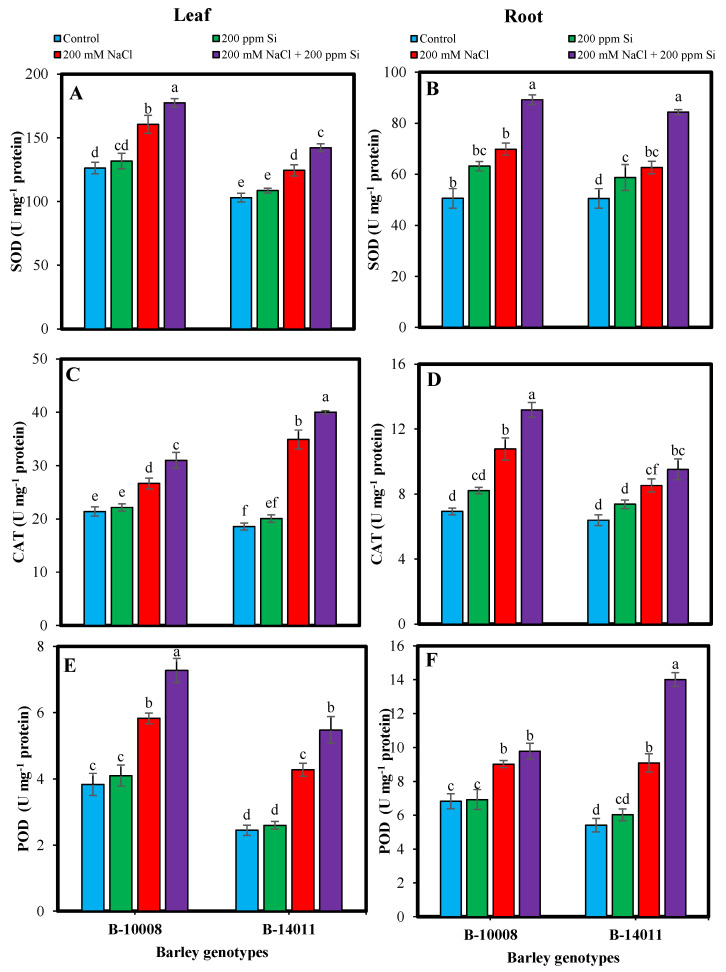
Superoxide dismutase (SOD) (**A**,**B**), catalase (CAT) (**C**,**D**) and peroxidase (POD) (**E**,**F**) (U mg^−1^ protein) contents in leaf and root of barley genotypes modulated by application of silicon through rooting media under salinity stress. The values represented by bars are means of four replicates ± SE. Different small letters (a–f) on bars denote significant difference at *p* ≤ 0.05 (Duncan’s multiple range test). Values sharing same letter within each subfigure are non-significant statistically.

**Figure 2:**
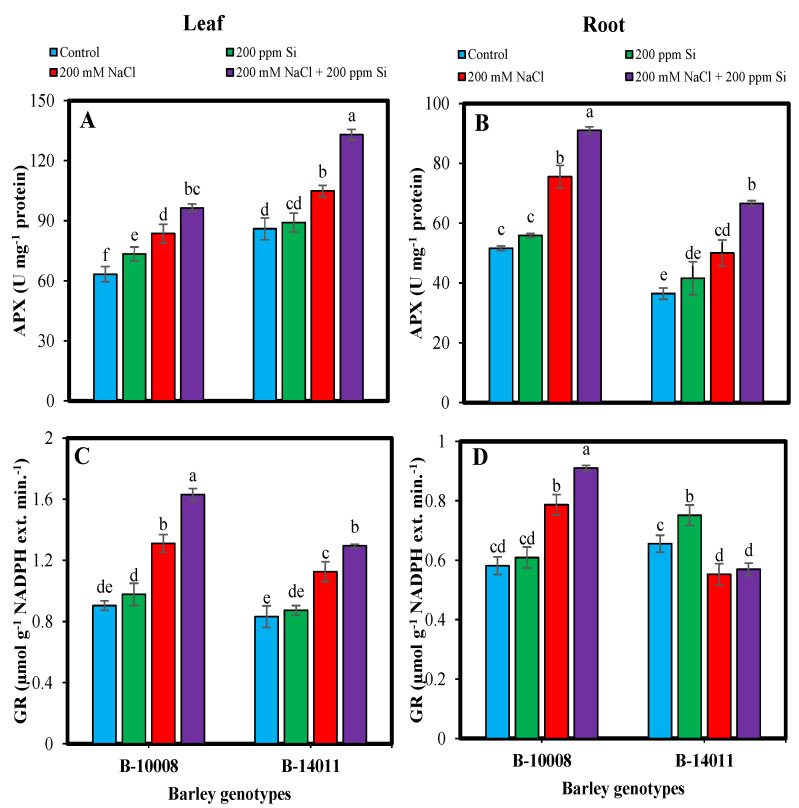
Ascorbate peroxidase (APX) (**A**,**B**) (Umg^−1^ protein) and glutathione reductase (GR) (**C**,**D**) (µmol g^−1^ NADPH ext. min^−1^) contents in leaf and root of barley genotypes modulated by application of silicon through rooting media under salinity stress. The values represented by bars are means of four replicates ± SE. Different small letters (a–e) on bars denote significant difference at *p* ≤ 0.05 (Duncan’s multiple range test). Values sharing same letter within each subfigure are non-significant statistically.

Si application (200 ppm) through rooting media further enhanced the activities of enzymatic antioxidants, especially under salinity stress. Maximum accumulation in SOD (14%, 34%), APX (26%, 33%) and POD (28, 54%) was found in leaves and roots of sensitive genotype (B-14011). Whereas the rooting application of Si under salinity stress in tolerant genotype (B-10008) resulted in the synthesis of higher leaf and root CAT (16%, 22%) and GR (24%, 15%) contents, respectively. The Si-mediated enhancement in the accumulation of enzymatic antioxidants was comparatively higher in genotype B-14011 comparable to B-10008 under salt stress after application of Si through rooting media (Figure 1 and Figure 2).

### 2.6. Non-Enzymatic Antioxidants

**Figure 3:**
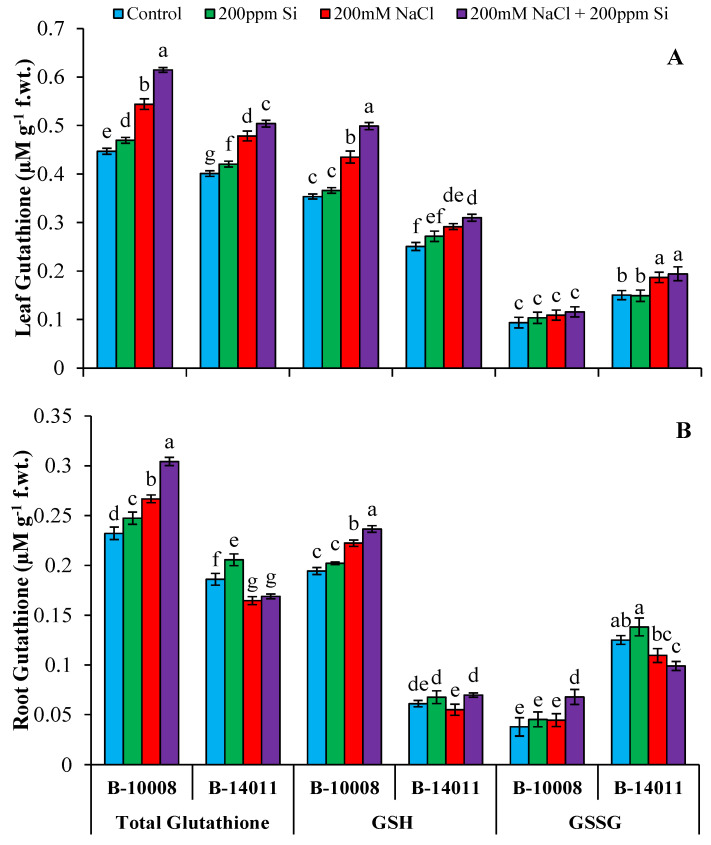
Glutathione (µmol g^−1^ f.wt.) contents in leaf (**A**) and root (**B**) of barley genotypes modulated by application of silicon through rooting media under salinity stress. The values represented by bars are means of four replicates ± SE. Different small letters (a–g) on bars denote significant difference at *p* ≤ 0.05 (Duncan’s multiple range test). Values sharing same letter within each subfigure are non-significant statistically. GSH = reduced glutathione, GSSG = oxidized glutathione, f.wt. = fresh weight.

**Figure 4:**
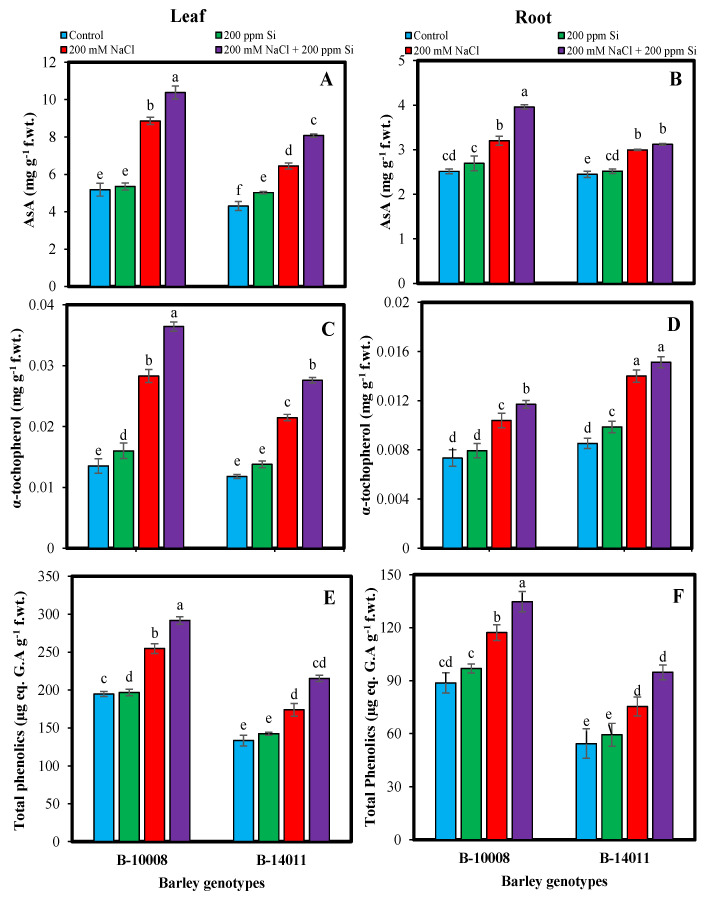
Ascorbic acid (AsA) (**A**,**B**) (mg g^−1^ f.wt.), α-tocopherol (**C**,**D**) (mg g^−1^ f.wt.) and total phenolics (**E**,**F**) (µg eq. GA g^−1^ f.wt.) contents in leaf and root of barley genotypes modulated by application of silicon through rooting media under salinity stress. The values represented by bars are means of four replicates ± SE. Different small letters (a–e) on bars denote significant difference at *p* ≤ 0.05 (Duncan’s multiple range test). Values sharing same letter within each subfigure are non-significant statistically.

The activities of non-enzymatic antioxidants, i.e., leaf and root glutathione (total, GSH, GSSG) (Figure 3), AsA, α-tocopherol and total phenolics (Figure 4) were significantly enhanced under salinity stress. Under salinity stress, maximum leaf and root total glutathione (21%, 14%) contents were observed in genotype B-10008, while root total glutathione contents of sensitive genotype was decreased (11%). Maximum leaf and root GSH (23%, 14%) and root GSSG (17%) contents were observed in genotype B-10008, while GSH and GSSG content in leaf and root of B-14011 were decreased to 10% and 12%, respectively, under salinity stress (Figure 3). Maximum leaf α-tocopherol (109%) contents were observed in B-10008, whereas, in the root the highest α-tocopherol (64%) contents were detected in B-14011 under salinity stress. Similarly, the genotypes B-14011 accumulated highest total phenolics [leaf (30%) and root (38%)] while B-10008 accumulated maximum AsA [leaf (78%) and root (27%)] contents in response to salty stress as compared to control plants (Figure 4).

Just like enzymatic antioxidants, the activities of non-enzymatic antioxidants were also enhanced after application of Si through rooting media in barley plants subjected to salinity stress, except for root GSSG contents, which were lowered under salinity stress in B-14011. The results revealed that as compared to saline treatment the genotypes B-10008 accumulated maximum leaf total glutathione (13%), GSH (15%) and GSSG (6%) after application of Si through rooting media. Similarly, maximum root total glutathione (14%) and GSSG (52%) contents were observed in B-10008 while root GSH (27%) contents were higher in B-14011 under salinity stress after Si application (Figure 3).

It has been observed that Si application under salinity stress more prominently enhanced α-tocopherol contents in leaf (29%) and root (13%) of genotype B-10008 while highest total phenolics contents in leaf (24%) and root (26%) was observed in B-14011 at same growing conditions. Similarly, the genotype B-14011 accumulated maximum leaf AsA (25%) while B-10008 showed highest root AsA (24%) contents after application of Si through rooting media under salinity stress (Figure 4).

**Figure 5:**
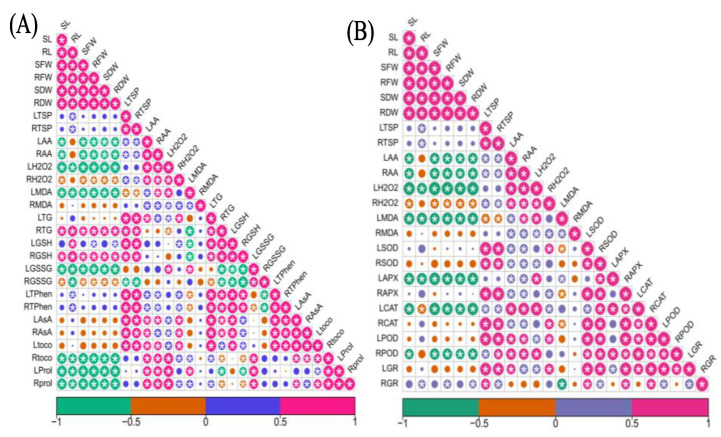
Pearson correlation analysis of different measured attributes of barley genotypes (B10008 and B14011) with non-enzymatic (**A**) and enzymatic (**B**) antioxidants modulated by application of silicon through rooting media under salinity stress. Asterisk (*) denotes significance of the interaction at *p* ≤ 0.05. Color of circles corresponds, i.e., Red: highly positive; Purple: slightly positive; Green: highly negative; Brown: slightly negative. Circle intensity or size shows the strength of correlation: stronger (Bigger) or weaker (Smaller). SL: shoot length; RL: root length; SFW: shoot fresh weight; RFW: root fresh weight; SDW: shoot dry weight; RDW: root dry weight; LTSP: leaf total soluble protein; RTSP: root total soluble protein; LAA: leaf free amino acids; RAA: root free amino acids; LH_2_O_2_: leaf hydrogen peroxide; RH_2_O_2_: root hydrogen peroxide; LMDA: leaf malondialdehyde; RMDA: root malondialdehyde; LTG: leaf total glutathione; RTG: root total glutathione; LGSH: leaf reduced glutathione; RGSH: root reduced glutathione; LGSSG: leaf oxidized glutathione; RGSSG: root oxidized glutathione; LTPhen: leaf total phenolics; RTPhen: root total phenolics; LAsA: leaf ascorbic acid; RAsA: root ascorbic acid; Ltoco: leaf α-tocopherol; Rtoco: root α-tocopherol; LProl: leaf proline; Rprol: root proline; LSD: leaf superoxide dismutase; RSOD: root superoxide dismutase; LAPX: leaf ascorbate peroxidase; RAPX: root ascorbate peroxidase; LCAT: leaf catalase; RCAT: root catalase; LPOD: leaf peroxidase: RPOD: root peroxidase: LGR: leaf glutathione reductase; RGR: root glutathione reductase.

The remarkable increase in the accumulation of proline was observed in both barley genotypes, especially in B-14011, as compared to B-10008 at 200 mM NaCl stress as compared to control treatment (Figure 5) as the former genotype accumulated maximum proline contents in leaves (872%) and roots (390%) under a salty environment. However, external fertigation of Si further boosted proline contents in both barley genotypes. Highest proline contents (leaf 40% and root 42%) were detected in genotype B-10008 after Si application under salt stress as compared to salt stress alone (Table 2).

### 2.7. Correlations

The correlation analysis of morphological attributes, TSP, TFAA, H_2_O_2_, and MDA with non-enzymatic and enzymatic antioxidants is presented in (Figure 5A,B). The data showed that there was a strong positive correlation among different morphological attributes. However, leaf and root TFAA, leaf H_2_O_2_ and leaf MDA had a strong negative relationship with morphological growth attributes (*p* ≤ 0.05) (Figure 5A,B).

Similarly, the relationship of leaf H_2_O_2_ and non-enzymatic antioxidants has a strong positive correlation with leaf GSSG, root tocopherol and leaf and root proline contents, while negative with leaf, and root AsA and leaf tocopherol while root H_2_O_2_, showed vice versa results (*p* ≤ 0.05). Root total glutathione and GSH also has a positive correlation with growth as well as root TSP contents (Figure 5A). However, among non-enzymatic antioxidants a positive relationship was found, except for root GSSG, which does not show any relationship with the majority of non-enzymatic antioxidants (Figure 5A).

Similarly, the correlation among leaf TSP, TFAA, H_2_O_2_ and MDA with enzymatic antioxidants also showed great variations (Figure 5B). TSP (Leaf and root) exhibited a strong positive correlation with leaf and root SOD, root APX and CAT, leaf PSD and GR while negative with leaf CAT. Leaf H_2_O_2_ exhibited positive relationship with leaf MDA, leaf APX, leaf CAT on the other hand it had negative relationship with leaf POD and GR. A positive correlation was found among enzymatic antioxidants with some exceptions on the behalf of leaf APX (Figure 5B).

### 2.8. Principal Component Analysis (PCA)

**Figure 6:**
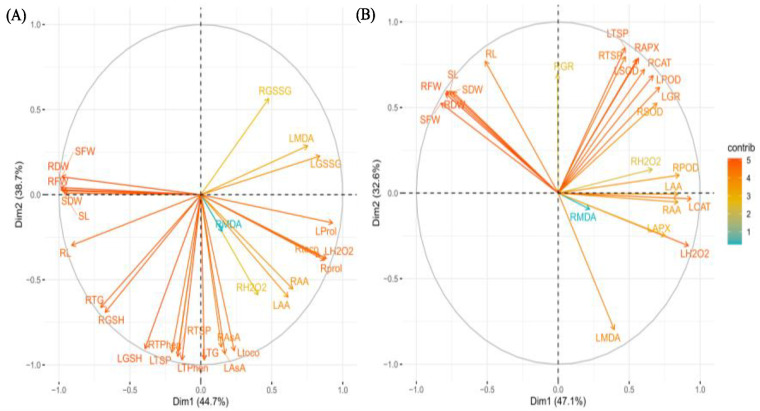
Principal component analysis (PCA) showing the relationship of different morphological, total soluble protein, amino acids, H_2_O_2_ and MDA contents with non-enzymatic (**A**) and enzymatic (**B**) antioxidants of barley genotypes modulated by application of silicon through rooting media under salinity stress. SL: shoot length; RL: root length; SFW: shoot fresh weight; RFW: root fresh weight; SDW: shoot dry weight; RDW: root dry weight; LTSP: leaf total soluble protein; RTSP: root total soluble protein; LAA: leaf free amino acids; RAA: root free amino acids; LH_2_O_2_: leaf hydrogen peroxide; RH_2_O_2_: root hydrogen peroxide; LMDA: leaf malondialdehyde; RMDA: root malondialdehyde; LTG: leaf total glutathione; RTG: root total glutathione; LGSH: leaf reduced glutathione; RGSH: root reduced glutathione; LGSSG: leaf oxidized glutathione; RGSSG: root oxidized glutathione; LTPhen: leaf total phenolics; RTPhen: root total phenolics; LAsA: leaf ascorbic acid; RAsA: root ascorbic acid; Ltoco: leaf α-tocopherol; Rtoco: root α-tocopherol; LProl: leaf proline; Rprol: root proline; LSD: leaf superoxide dismutase; RSOD: root superoxide dismutase; LAPX: leaf ascorbate peroxidase; RAPX: root ascorbate peroxidase; LCAT: leaf catalase; RCAT: root catalase; LPOD: leaf peroxidase: RPOD: root peroxidase: LGR: leaf glutathione reductase; RGR: root glutathione reductase.

The results of Pearson’s correlation analysis were further confirmed by PCA-Biplot that showed that synthesis of antioxidants in barley has a direct relationship with plant growth, which on the other hand, is compromised by the excessive production of ROS and lipid peroxidation (Figure 6A,B). To infer the relationship of enzymatic and non-enzymatic antioxidants with morphological attributes (SL, RL, SFW, SDW, RFW, RDW), H_2_O_2_ and MDA contents, we performed PCA analysis. The two components of PCA, i.e., PC1 and PC2 were represented as Dim1 and Dim2, respectively. The cumulative variance in Dim1 and Dim2 for non-enzymatic and enzymatic antioxidants accounts for 83% and 80%, respectively (Figure 6A,B). The both PCAs exhibited that the majority of non-enzymatic and all enzymatic antioxidants, ROS (H_2_O_2_) and lipid peroxidation (MDA) are found in Dim1. So the first component may be named as antioxidant scavenger and salinity tolerance, while in Dim2, all the growth related morphological attributes are presented (Figure 6A,B).

The angle between LH_2_O_2_ and LProl; LMDA and LGSSG; RH_2_O_2_ and RMDA; LMDA and LGSSG (Figure 6A) and SFW, SDW, RFW, RDW, SL and RL and in PCA (Figure 6A,B) showed that there exist a strong positive. It has been observed that RH_2_O_2_ and RGSSG exhibited no relationship between these two components. Similarly, the data presented in Figure 6B revealed that there is a strong positive correlation between LH_2_O_2_ and LAPX; LTSP, LSOD, LPOD, LGR, RTSP, RAPX, RCAT, RAPX and RSOD, RH_2_O_s_ and RPOD and LH_2_O_2_ and LCAT, while the angle between LH_2_O_2_ and morphological attributes showed that a strong negative correlation exists. The representation of RMDA showed that it had a little contribution in both PCAs (Figure 6A,B).

## 3. Discussion

Salinity stress seriously hampers plant’s growth and development through a reduction in the photosynthetic process, upsetting ionic equilibrium and enhancing oxidative damage [31]. The impact of salinity on a plant’s growth is a time dependent process, summarized in a two-phase model: (i) rapid phase ascribed to water deficit conditions (osmotic); and (ii) slow phase caused by the accumulation of ions to toxic levels (ion-specific) causing negative effects on plant’s physio-biochemical activities [32].

Salinity-induced reduction in biomass accumulation and plant height was observed in this experiment, however, this effect was more pronounced on salt sensitive barley genotype (B-14011) as compared to a salt tolerant one (B-10008). The decrease in biomass and lengths of plants was mainly the result of an excessive buildup of Na^+^ and Cl^−^ [4] due to a disturbance in nutrient uptake [32] and a reduction in te photosynthetic process, which is directly related to biomass accumulation [33]. Salinity-induced reduction in morphological attributes has been previously observed in maize [34], wheat [35], canola [36], and barley [37].

The application of Si through rooting media showed a positive effect on growth of barley plants. Si-mediated increase in biomass and plant height under salinity is attributed to improved nutrient balance, reduced uptake of Na^+^, higher photosynthetic rates and efficient detoxification of excessive ROS via enhanced antioxidant activity [18,34]. The Si-mediated enhancement in growth under salinity stress was previously reported by Yan et al. [38] in wheat, Ahmad et al. [12] in mung bean, Laifa et al. [39] in barley and Raza et al. [34] in maize.

The over-accumulation of H_2_O_2_ in cellular system under salinity results in fatty acid oxidation leading to membrane damage and electrolyte leakage, as was observed as enhanced MDA levels under salt stress, especially in B-14011. This salinity-induced production of ROS (oxidative stress) harms lipids, proteins, carbohydrates and nucleic acid leady to cell death [40]. On the other hand, Si application through external means in salt treated plants ameliorated extra ROS (H_2_O_2_) and caused reduction in lipid peroxidation (MDA). The main source of ROS is mitochondria and chloroplasts where it is accumulated during electron transport processes [41], which can start lipid peroxidation in the cell [42]. AbdElgawad et al. [43] reported that 150 mM NaCl enhanced the production of H_2_O_2_ and enhanced lipid peroxidation (higher MDA level) in maize. The Si application tends to maintain the metabolism of plant to an optimum level by decreasing ROS production, lowering lipid peroxidation, maintaining integrity of membranes and reducing leakage of electrolyte from cytosol in many crops like wheat [29], rice [38], Basil [24] and sunflower [44] and maize [30].

The immediate response of plants to the overproduction of ROS is enhancement in the synthesis of TSPs, TFAAs. Similarly, the activation of enzymatic and non-enzymatic antioxidants is also enhanced in order to efficiently scavenge excessive ROS [12,43]. As compared to B-14011 the genotype B-10008 synthesized higher TSP contents under salt stress, while the accumulation of TFAA was higher in B-14011 than B-10008. However, imposition of Si augmented TSP contents in leaves and roots of barley plants. This enhancement in TSP accumulation indicate that plant’s endogenous defense system was boosted under salinity stress [45,46].

The enzymatic antioxidant (CAT, POD, SOD, APX, and GR) activities were boosted under salinity stress. The accumulation of CAT and POD was higher in B-14011, and displaying that increase in H_2_O_2_ levels enhanced these antioxidants. The salinity induced enhancement in the activities of enzymatic antioxidant, i.e., CAT, POD, SOD, APX and GR is reported in maize [30], sunflower [44], wheat [29] and alfalfa [47]. The stimulation in the synthesis of non-enzymatic antioxidants (AsA, α-tocopherol, total phenolics, glutathione and proline) in different plant tissues of barley genotypes was significantly enhanced under salinity stress. Comparatively, the non-enzymatic antioxidant activities were higher in B-10008 as compared to B-14011, when compared to their respective controls under salt stress. It has been observed that many plants enhance the activities of non-enzymatic antioxidants to safeguard the cellular structures from ROS-induced oxidative damage under salinity stress [24,48,49].

The application of Si through external means boosted the salinity-induced scavenging of ROS through antioxidant defense system in both barley genotypes. The possible mechanism of Si-induced boosted defense system under salinity stress is due to reduction in Na^+^ uptake; increased K^+^ absorption, improved water status, enhanced water retention capacity and finally limiting ROS production [50]. The similar results were previously reported in millet [51], maize [52], alfalfa [53] and wheat [54]. This study also uncovered the fact that Si application enhanced the activities of enzymatic (CAT, POD, SOD, APX and GR) and non-enzymatic (AsA, α-tocopherol, total phenolics, glutathione and proline) antioxidants under salt stress. The sensitive barley genotype (B-14011) showed a comparatively better antioxidant response analogous to a tolerant genotype (B-10008) under salinity stress.

The Si-mediated enhancement in antioxidant activities reduced the oxidative damages posed by ROS, thus lowering lipid peroxidation and conserving membrane permeability [31,55]. Although results clearly demonstrate that there is a clear difference in antioxidant response in Si-treated and non-Si treated barley plants, a gap still exists in clarifying the interaction of exogenously applied Si and the antioxidant battery system of plants. Under salinity stress, Si application through external means reduced the uptake of Na^+^ by stimulating the root plasma membrane H^+^-ATPase activity, which can possibly lower ROS thus enhancing salt tolerance aided by efficient antioxidant defense system [56,57].

Enhanced proline accumulation under stressful conditions is regarded as a defense response of plants to a specific stress [58]. In this experiment the proline contents was considerably enhanced in both barley genotypes, however the response of B-14011 was exponentially high as compared to B-10008 genotypes, whereas presence of Si in media further boosted proline synthesis under salinity stress. It has been generally accepted that proline accumulation is a stress adoptive strategy in tolerant plant species [59]. Yet, proline over-accumulation under salt stress cannot be regarded as a permanent bench-mark for salt tolerance as there are many reports that concluded that sensitive genotypes/varieties had accumulated much higher proline when compared to tolerant ones [60,61]. This proline over accumulation in sensitive genotypes is an indication of salt injury [62]. Results of this experiment showed that Si application lowered proline contents of both barley genotypes. Previously, Tuna et al. [63] in wheat, Soylemezoglu et al. [64] in grape, Yin et al. [65] in sorghum and Gunes et al. [66] in barley have reported similar results showing that Si application can reduce the proline contents under salt stress.

## 4. Materials and Methods

The experiment was designed and conducted at Bio-Park of Institute of Pure and Applied Biology, Bahauddin Zakariya University, Multan, Pakistan during two successive seasons 2017–2018 and 2018–2019. Surface sterilized (Sodium hypochlorite solution) seeds of two barley genotypes; B-14011 and B-10008 were grown in pots weighing 8 kg of river sand arranged in a completely randomized design (CRD) with four replicates of each treatment. The pots were arranged in two sets; 1st non-saline (irrigated with water + Hoagland nutrient solution) and second saline (irrigated with 200 mM NaCl + Hoagland nutrient solution). The 50% pots from non-saline and 50% from saline set were irrigated with 200 ppm Si (K_2_SiO_3_.2H_2_O) solution through rooting media when seedlings were two weeks old. During the third week of germination, 50% pots of each genotype were irrigated with 200 mM NaCl solution (saline), while the remaining 50% were irrigated with tap water (non-saline). Hoagland and Arnon [67] nutrient solution was supplied to plants to fulfill their nutrient requirements.

### 4.1. Morphological Attributes

The plants were carefully uprooted sixty days after germination, washed with distilled water; placed in plastic bags immediately and the length and weight of roots and shoots were recorded, while dry weights were recorded after drying the samples for 96 h at 70 °C.

### 4.2. Estimation of Biochemical Attributes

For the estimation of proteins, amino acids and antioxidants the leaf and root samples were homogenized in 50 mM Na^+^-phosphate buffer at 4 °C. The material was then centrifuged for 12 min at 15,000 rpm and supernatant was removed carefully to be used for biochemical assay.

#### 4.2.1. Total Soluble Proteins (TSP)

The 0.1 mL of supernatant was poured in test tubes containing 5 mL of Bradford reagent. The reading was taken at 595 nm using uv-vis spectrophotometer (U-2900 Hitachi) after 15 mints of incubation at room temperature [68].

#### 4.2.2. Total Free Amino Acids (TFAA)

For TFAA estimation, 0.5 mL of supernatant was added to 0.5 mL ninhydrin (2%) and 0.5 mL pyridine (10%) solution in test tubes and was water bathed for 30 min at 100 °C. After cooling, the volume was raised to 25 mL with distilled water and absorbance was recorded at 570 nm with spectrophotometer [69].

### 4.3. Enzymatic Antioxidants

#### 4.3.1. Superoxide Dismutase (SOD)

The SOD activities in leaves and roots were determined through quantifying the inhibition in photo reduction in nitrobluetetrazolium (NBT), the protocol devised by Beauchamp and Fridovich [70]. Reaction solution was prepared by mixing: (i) 75 µL of NBT; (ii) 20 µL of riboflavin; (iii) 130 mL of L-methionine; and (iii) 100 µL of Na_2_EDTA into sodium phosphate buffer. Reaction solution (2.725 mL) was mixed with of dH_2_O (0.25 mL) and 50 µL enzyme extract (supernatant) into a glass beaker and was kept in the dark. A similar set of beakers was prepared and placed at light conditions of 4000 lux for 15 min. The absorbance in the dark adopted and illuminated samples was recorded at 560 nm using spectrophotometer.

#### 4.3.2. Peroxidase (POD) and Catalase (CAT)

The reaction solution for POD contained 100 µL 30 mM H_2_O_2_, 100 µL guaiacol and 100 µL of enzyme extract (supernatant) into 2.7 mL sodium phosphate buffer. However, for the estimation of CAT activity, the same reaction solution that was used for POD (except guaiacol) was used. The absorbance of POD and CAT samples was observed on time scan (0–60 s) at 470 and 240 nm, respectively, using spectrophotometer [71].

#### 4.3.3. Ascorbate Peroxidase (APX)

The activity of APX was determined using Nakano and Asada [72] methodology. The reaction solution contained 100 µL ascorbate solution (10 mM), 100 µL H_2_O_2_ (30%) and 100 µL enzyme extract (supernatant) into 2.7 mL of sodium phosphate buffer. After a gentle shake, the absorbance was read at 290 nm with on time scan (0–60 s) using a spectrophotometer.

#### 4.3.4. Glutathione Reductase (GR)

To assay GR, the reaction solution (2 mL) consisted of 100 µL NADPH (0.15 mM), 100 µL GSSG (0.5 mM), 700 µL dH_2_O and 100 µL of supernatant into 1000 µL of potassium phosphate buffer containing Na2EDTA (2 mM). The addition of NADPH started the oxidation reaction. The NADPH oxidation in each sample was assessed at 340 nm using spectrophotometer [73].

### 4.4. Non-Enzymatic Antioxidants

#### 4.4.1. Total Phenolics

Fresh plant samples were ground in 80% acetone and were centrifuged at 8000 rpm for 10 min. The supernatant (100 µL) was reacted with 100 µL Folin Ciocalteu’s phenol reagent and 5 mL of Na_2_CO_3_ (20%). The volume was then raised to 10 mL with dH_2_O. After vigorous shaking, absorbance of samples was observed at 750 nm [74].

#### 4.4.2. Ascorbic acid (AsA)

For AsA estimation, plant samples were completely homogenized in 6% TCA solution and were centrifuged at 8000 rpm for 10 min. The reaction solution consisted of 4 mL supernatant, 2 mL of 2% dinitrophenylhydrazine and one drop of thiourea (10%) solution, mixed in test tubes. The reaction solutions were then water bathed for 20 min at 100 °C followed by cooling at room temperature. The test tubes were then shifted to ice and 5 mL of 80% H_2_SO_4_ was mixed slowly in these tubes. The absorbance of samples was recorded at 530 nm [75].

#### 4.4.3. α-Tocopherol (Vitamin E)

The α-tocopherol contents were assayed by grinding plant samples in a mixture of petroleum ether:ethanol (2:1.6 *v*/*v*). The homogenized material was centrifuged at 8000 rpm for 20 min. The supernatant (1 mL) was mixed with 200 µL of 2% 2,2-dipyridyl and placed in dark adopted incubation for 5 min till the final red coloration of solution. The volume of solution was raised to 4 mL with dH_2_O and absorbance of the samples was read at 520 nm [76].

#### 4.4.4. Glutathione Contents

Plant samples were homogenized in 10% TCA solution, centrifuged for 12 min at 12,000 rpm and supernatant was removed for the estimation of total glutathione, reduced glutathione (GSH) and oxidized glutathione (GSSG) contents. To assay total glutathione, 200 µL supernatant was added 600 µL sodium phosphate buffer (125 mM, pH 7.5), 1 mL GR enzyme (10 units mL^−1^), 200 µL DNTB (6 mM) and 1 mL of NADPH (0.3 mM) solution. For GSH assay, 200 µL supernatant was added to 1.4 mL sodium phosphate buffer (125 mM, pH 7.5) and 200 µL DNTB (6 mM). The mixtures were water bathed with stirring at 30 °C for 10 min followed by an immediate ice bath before the reading was taken. The absorbance for total glutathione and GSH was taken at 412 nm [77]. The GSSG concentration in samples was worked out by subtracting GSH from total glutathione.

#### 4.4.5. Proline

For proline estimation, plant samples (0.5 g) were grinded in 10 mL 3% sulfosalicylic acid solution and were filtrated with Whatman filter paper. A total of 2 mL of extract sample was added to 2 mL ninhidrin and 2 mL of glacial acetic acid solution in test tubes, which were water bathed at 100 °C for 60 min followed by immediate cooling in ice. After cooling, 4.0 mL of toluene was poured into these test tubes, mixed vigorously and kept at room temperature until two layers were formed. The absorbance of the upper colored layer was taken at 520 nm [78].

### 4.5. Hydrogen Peroxide (H_2_O_2_) and Malondialdehyde (MDA)

To assay H_2_O_2_ contents, the plant material (0.25 g) was homogenized in 5 mL TCA (0.1%) solution and centrifuged for 15 min at 12,000 rpm. The supernatant (0.5 mL) was mixed with 0.5 mL sodium phosphate buffer and 1 mL of potassium iodide (KI) solution in test tubes. Test tubes were vortexed and absorbance was read at 390 nm [79].

The MDA contents were estimated using Heath and Packer [80] methodology. Then, 1 mL of supernatant (same as used in protein estimation) was mixed to 1 mL TBA (0.5%) solution prepared in 20% TCA solution in test tubes and were water bathed for 30 min at 95 °C. The tubes were then ice bathed for 5 min followed centrifugation at 6000 rpm. The absorbance was recorded at 532 nm and 600 nm. The extension coefficient (155 mM^−1^ cm^−1^) was used for MDA contents calculation.

### 4.6. Statistical Analysis

Three-way ANOVA was subjected to data using SPSS-20.0 (SPSS Inc. Chicago, IL, USA). The genotypes (G), salinity (S) and silicon (Si) were used as fixed factors. The Duncan’s Multiple Range Test (DMRT) at *p* < 0.05 probability was subjected to observe the difference among means.

## 5. Conclusions

Salinity stress reduced the growth by enhancing the levels of ROS (H_2_O_2_) and MDA in barley plants. However, the plants responded to salinity stress by osmotic adjustment, activating the antioxidant defense system and initiating stress-induced signaling pathway. Along with the plant’s internal regulatory mechanism, the application of Si adds the plant’s protective machinery in the alleviation of stress by regulating several metabolic pathways for detoxification of excessive ROS. This study concluded that application of Si can boost up barley plant’s metabolism by protecting cell’s bio molecules through enhanced production of enzymatic (SOD, APX, CAT, POD and GR) and non-enzymatic (phenolics, α-tocopherol, AsA, glutathione, proline) antioxidants and by limiting lipid peroxidation (MDA) and ROS (H_2_O_2_) production under salinity stress. As such, the application of Si through rooting media should be used as an early and fast remedy to mitigate salinity stress in plants.

## Figures and Tables

**Table 1:** Morphological attributes of barley genotypes modulated by rooting application of silicon under salinity stress.

Genotype	Treatment	Shoot Length (cm)	Root Length (cm)	Shoot Fresh Weight (g)	Shoot Dry Weight (g)	Root Fresh Weight (g)	Root Dry Weight (g)
**B-10008**	Control	68.53 ± 3.72 ^b^	52.04 ± 1.46 ^b^	124.13 ± 1.65 ^b^	15.73 ± 0.72 ^b^	70.90 ± 3.49 ^ab^	7.53 ± 0.11 ^ab^
	200 ppm Si	81.40 ± 1.51 ^a^	61.13 ± 1.87 ^a^	146.80 ± 2.63 ^a^	19.34 ± 0.54 ^a^	77.91 ± 1.85 ^a^	8.34 ± 0.48 ^a^
	200 mM NaCl	50.14 ± 1.59 ^d^	42.13 ± 2.42 ^c^	86.52 ± 2.43 ^e^	9.74 ± 0.73 ^d^	49.63 ± 1.69 ^c^	5.12 ± 0.32 ^e^
	200 mM NaCl + 200 ppm Si	63.71 ± 1.32 ^c^	53.04 ± 2.07 ^b^	105.33 ± 3.10 ^c^	14.50 ± 1.14 ^bc^	67.62 ± 2.35 ^b^	6.99 ± 0.25 ^bc^
**B-14011**	Control	51.14 ± 0.67 ^d^	30.21 ± 1.68 ^d^	96.04 ± 4.10 ^d^	10.71 ± 0.91 ^d^	53.24 ± 4.71 ^c^	5.74 ± 0.35 ^de^
	200 ppm Si	61.12 ± 1.83 ^c^	32.10 ± 3.58 ^d^	107.62 ± 2.05 ^c^	12.73 ± 0.46 ^c^	62.42 ± 2.13 ^b^	6.32 ± 0.22 ^cd^
	200 mM NaCl	20.31 ± 0.99 ^f^	13.04 ± 1.20 ^e^	25.53 ± 1.26 ^g^	2.90 ± 0.40 ^e^	7.73 ± 0.56 ^d^	0.77 ± 0.09 ^f^
	200 mM NaCl + 200 ppm Si	27.33 ± 1.22 ^e^	18.02 ± 0.75 ^e^	34.03 ± 1.65 ^f^	4.29 ± 0.34 ^e^	10.35 ± 0.32 ^d^	1.00 ± 0.01 ^f^

Values are means ± SE (*n* = 4), letters (a–g) represent significant difference at *p* ≤ 0.05 (Duncan’s multiple range test). Values sharing the same letter within each column are non-significant statistically.

**Table 2:** Total soluble protein (TSP), total free amino acid (TFAA), hydrogen peroxide (H_2_O_2_), malondialdehyde (MDA) and proline (µM f.wt.) contents in leaf and root of barley genotypes modulated by rooting application of silicon under salinity stress.

		Leaf				
Genotype	Treatments	TSP (mg g^−1^ f.wt.)	TFAA (mg g^−1^ f.wt.)	H_2_O_2_ (µmol g^−1^ f.wt.)	MDA (nmol g^−1^ f.wt.)	Proline (µM f.wt.)
**B-10008**	Control	29.03 ± 0.30 ^e^	7.56 ± 0.41 ^c^	0.22 ± 0.017 ^f^	29.04 ± 0.64 ^c^	3.92 ± 0.52 ^f^
	200 ppm Si	32.21 ± 0.75 ^c^	7.69 ± 0.08 ^c^	0.26 ± 0.006 ^e^	28.52 ± 0.76 ^c^	4.04 ± 0.71 ^f^
	200 mM NaCl	35.53 ± 0.98 ^b^	8.96 ± 0.33 ^b^	0.59 ± 0.028 ^c^	33.01 ± 0.77 ^b^	35.72 ± 3.08 ^c^
	200 mM NaCl + 200 ppm Si	41.62 ± 1.06 ^a^	8.43 ± 0.40 ^b^	0.38 ± 0.007 ^d^	19.63 ± 1.18 ^d^	25.55 ± 1.19 ^d^
**B-14011**	Control	24.24 ± 0.32 ^g^	6.07 ± 0.22 ^d^	0.19 ± 0.005 ^f^	33.80 ± 1.79 ^b^	14.76 ± 1.10 ^e^
	200 ppm Si	26.90 ± 0.78 ^f^	6.19 ± 0.07 ^d^	0.24 ± 0.018 ^ef^	27.04 ± 1.39 ^c^	16.77 ± 3.41 ^e^
	200 mM NaCl	29.62 ± 1.38 ^de^	10.50 ± 0.15 ^a^	0.91 ± 0.028 ^a^	43.61 ± 0.63 ^a^	178.09 ± 3.10 ^a^
	200 mM NaCl + 200 ppm Si	31.34 ± 0.30 ^cd^	9.04 ± 0.10 ^b^	0.77 ± 0.029 ^b^	35.43 ± 2.11 ^b^	143.44 ± 2.11 ^b^
		**Root**				
**Genotype**	**Treatments**	**TSP**	**TFAA**	**H_2_O_2_**	**MDA**	**Proline**
**B-10008**	Control	11.04 ± 0.30 ^c^	1.20 ± 0.06 ^d^	0.18 ± 0.020 ^de^	11.70 ± 1.01 ^bc^	2.15 ± 0.13 ^d^
	200 ppm Si	11.72 ± 0.41 ^bc^	1.25 ± 0.07 ^d^	0.19 ± 0.007 ^de^	10.80 ± 0.97 ^cd^	3.01 ± 0.24 ^d^
	200 mM NaCl	14.91 ± 0.77 ^a^	1.48 ± 0.13 ^bc^	0.45 ± 0.028 ^a^	13.75 ± 1.09 ^ab^	9.45 ± 0.36 ^b^
	200 mM NaCl + 200 ppm Si	16.03 ± 0.63 ^a^	1.38 ± 0.08 ^cd^	0.35 ± 0.029 ^b^	8.66 ± 0.54 ^de^	6.65 ± 0.91 ^bc^
**B-14011**	Control	6.73 ± 0.38 ^e^	0.93 ± 0.04 ^e^	0.26 ± 0.041 ^c^	8.58 ± 0.58 ^de^	3.15 ± 0.16 ^d^
	200 ppm Si	7.35 ± 0.57 ^e^	0.88 ± 0.07 ^e^	0.15 ± 0.013 ^e^	7.99 ± 1.00 ^e^	3.85 ± 0.26 ^cd^
	200 mM NaCl	8.82 ± 0.34 ^d^	1.88 ± 0.05 ^a^	0.41 ± 0.013 ^a^	14.36 ± 1.65 ^a^	18.12 ± 3.19 ^a^
	200 mM NaCl + 200 ppm Si	12.74 ± 0.67 ^b^	1.59 ± 0.06 ^b^	0.24 ± 0.022 ^cd^	7.84 ± 0.49 ^e^	15.43 ± 1.52 ^a^

Values are means ± SE (*n* = 4), letters (a–e) represent significant difference at *p* ≤ 0.05 (Duncan’s multiple range test). Values sharing same letter within each column are non-significant statistically.

## Data Availability

Not applicable.
