# Correction: Akhter et al. Silicon-Induced Mitigation of NaCl Stress in Barley (*Hordeum vulgare* L.), Associated with Enhanced Enzymatic and Non-Enzymatic Antioxidant Activities. *Plants* 2022*, 11*, 2379

**DOI:** 10.3390/plants15132048

**Published:** 2026-07-02

**Authors:** Muhammad Salim Akhter, Sibgha Noreen, Ume Ummara, Muhammad Aqeel, Nawishta Saleem, Muhammad Mahboob Ahmed, Seema Mahmood, Habib-ur-Rehman Athar, Mohammed Nasser Alyemeni, Prashant Kaushik, Parvaiz Ahmad

**Affiliations:** 1 Institute of Botany, Bahauddin Zakariya University, Multan 60800, Pakistan; msalimakhter82@gmail.com (M.S.A.); nawish_s@yahoo.com (N.S.); seemamehmood@bzu.edu.pk (S.M.); habibathar@bzu.edu.pk (H.-u.-R.A.); 2 Department of Botany, The Islamia University of Bahawalpur, Rahim Yar Khan Campus, Rahim Yar Khan 64200, Pakistan; umeummara95@gmail.com; 3 State Key Laboratory of Grassland Agro-Ecosystems, School of Life Science, Lanzhou University, Lanzhou 730000, China; asghar16@lzu.edu.cn; 4 Institute of Chemical Science, Bahauddin Zakariya University, Multan 60800, Pakistan; mahboobahmed@bzu.edu.pk; 5 Botany and Microbiology Department, King Saud University, Riyadh 11451, Saudi Arabia; mnyemeni@ksu.edu.sa; 6 Independent Researcher, 46022 Valencia, Spain; prakau@doctor.upv.es; 7 Department of Botany, GDC, Pulwama 192301, Jammu and Kashmir, India

In the publication [1], there was an error regarding the affiliation for Prashant Kaushik. The updated affiliation should be: Independent Researcher, 46022 Valencia, Spain.

The authors state that the scientific conclusions are unaffected. This correction was approved by the Academic Editor. The original publication has also been updated.
